# Nuclear functions and subcellular trafficking mechanisms of the epidermal growth factor receptor family

**DOI:** 10.1186/2045-3701-2-13

**Published:** 2012-04-20

**Authors:** Ying-Nai Wang, Mien-Chie Hung

**Affiliations:** 1 Department of Molecular and Cellular Oncology, The University of Texas MD Anderson Cancer Center, Houston, TX, 77030, USA; 2 Center for Molecular Medicine and Graduate Institute of Cancer Biology, China Medical University, Taichung, Taiwan

**Keywords:** EGFR family receptors, Nuclear translocation, Subcellular trafficking

## Abstract

Accumulating evidence suggests that various diseases, including many types of cancer, result from alteration of subcellular protein localization and compartmentalization. Therefore, it is worthwhile to expand our knowledge in subcellular trafficking of proteins, such as epidermal growth factor receptor (EGFR) and ErbB-2 of the receptor tyrosine kinases, which are highly expressed and activated in human malignancies and frequently correlated with poor prognosis. The well-characterized trafficking of cell surface EGFR is routed, via endocytosis and endosomal sorting, to either the lysosomes for degradation or back to the plasma membrane for recycling. A novel nuclear mode of EGFR signaling pathway has been gradually deciphered in which EGFR is shuttled from the cell surface to the nucleus after endocytosis, and there, it acts as a transcriptional regulator, transmits signals, and is involved in multiple biological functions, including cell proliferation, tumor progression, DNA repair and replication, and chemo- and radio-resistance. Internalized EGFR can also be transported from the cell surface to several intracellular compartments, such as the Golgi apparatus, the endoplasmic reticulum, and the mitochondria, in addition to the nucleus. In this review, we will summarize the functions of nuclear EGFR family and the potential pathways by which EGFR is trafficked from the cell surface to a variety of cellular organelles. A better understanding of the molecular mechanism of EGFR trafficking will shed light on both the receptor biology and potential therapeutic targets of anti-EGFR therapies for clinical application.

## Review

### Introduction

Receptor tyrosine kinases (RTKs), which contain an extracellular ligand binding domain, a transmembrane domain, and an intracellular tyrosine kinase domain, mediate cellular signal transduction by extracellular ligand binding. The epidermal growth factor receptor (EGFR) family of RTKs consists of four members: EGFR/ErbB-1/HER-1, ErbB-2/HER-2/neu, ErbB-3/HER-3, and ErbB-4/HER-4, and all except ErbB-3 are associated with tyrosine kinase activity. Upon ligand binding, EGFR family proteins dimerize by receptor homo-dimerization or hetero-dimerization and subsequently activate tyrosine kinase activity. Activated EGFR family receptors then trigger a myriad of downstream signaling pathways, such as phosphatidylinositol-3 kinase, mitogen-activated protein kinase, signal transducer and activator of transcription (STAT), phospholipase C, and the modulation of calcium channels. These downstream signaling activities regulate proliferation, mobility, and differentiation in many different cell types [1-4].

All but ErbB-4 of the EGFR family of proteins are expressed and/or constitutively activated in human tumors of epithelial origin. This expression leads to aggressive tumor behavior, including cancer initiation, increased tumor growth/progression, poor patient outcome, metastasis, and chemo-resistance [5-8]. Unlike the other EGFRs, the oncogenic role of ErbB-4 in breast cancer is unclear since it appears to be correlated with prolonged patient survival and tumor growth suppression [9,10]. Accordingly, EGFR family receptors have been considered as effective targets for anti-cancer therapies. Both ectodomain-binding monoclonal antibodies and small-molecule tyrosine-kinase inhibitors (TKIs) targeting EGFR and ErbB-2 have been developed, and many of them are approved by the Food and Drug Administration.

Interestingly, in addition to their positions in traditional signaling cascades, numerous evidence to date from different groups indicate a unique translocation and the associated biological functions of the EGFR family receptors, by which they can be shuttled from the cell surface to the nucleus [11-35], termed membrane receptors in the nucleus (MRIN) [36]. The molecular mechanism underlying the cell surface membrane-to-nucleus trafficking of EGFR has been documented recently [37,38]. In this review, we will summarize recent discoveries in the MRIN field and discuss the subcellular trafficking pathways of the EGFR family proteins from the cell surface to a variety of cellular organelles, including the Golgi apparatus, the endoplasmic reticulum (ER), the mitochondria, as well as the nucleus.

### Discovery and current understandings of MRIN

#### Nuclear EGFR detection and clinical relevance

Nuclear expression of EGFR was first detected in hepatocytes during regeneration [39-42]. A full-length form of cell surface EGFR has been shown to be translocated to the nucleus, where evidence suggests that it is involved in transcriptional regulation, cell proliferation, DNA replication, DNA repair, and chemo- and radio-resistance [43-48]. In addition to ligand-dependent mechanisms, EGFR transport to the nucleus has also been associated with DNA damage events, including ionizing radiation, ultraviolet irradiation, cisplatin treatment, oxidative stress, heat treatment, and treatment with cetuximab/C225, a monoclonal anti-EGFR antibody [49-52]. Nuclear EGFR has been associated with poor clinical prognosis in diverse cancer types, including breast cancer, ovarian cancer, and oropharyngeal and esophageal squamous cell carcinomas [53-58].

#### Nuclear EGFR in therapeutic resistance

Nuclear EGFR has been shown to contribute to resistance to various cancer therapies, such as radiation, cisplatin, and cetuximab [59-61]. Moreover, dasatinib, a potent Src inhibitor, can block cetuximab- and radiation-induced EGFR nuclear translocation in head and neck squamous cell carcinoma [62]. Another report showed that lapatinib, a dual TKI of EGFR and HER2, inhibits the nuclear transport of EGFR and HER2 and sensitizes cancer cells to fluoropyrimidine by downregulating thymidylate synthase, which is frequently overexpressed in fluoropyrimidine-resistant cancer cells [63]. Recently, two related papers demonstrated that nuclear EGFR confers acquired resistance to an EGFR-TKI, gefitinib, by increasing the expression of breast cancer-resistant protein (BCRP), which is an ATP-binding cassette transporter that pumps anti-cancer drugs out of cells [17,64]. Combined evidence to date suggests that nuclear localization of EGFR contributes to therapeutic resistance to EGFR-targeting treatments.

#### Nuclear translocation of EGFR variants

EGFRvIII, a constitutively activated EGFR type III variant, was first identified in the nucleus in hormone-refractory prostate cancer and associated with poor patient survival [65]. EGFRvIII has also been detected in the nucleus of normal glial cells and primary glioblastomas, where it forms an oncogenic complex with STAT3 to mediate EGFRvIII-dependent glial transformation [26]. Another report showed that the nuclear EGFRvIII-STAT3 complex can activate cyclooxygenase-2 (COX-2) gene expression in glioblastoma cells [30]. Interestingly, an in-frame splicing EGFR variant that lacks multiple domains, called mLEEK, has recently been reported to function as a transcription factor in the nucleus [16].

#### Nuclear translocation of ErbB-2

The level of ErbB-2/neu has been shown increase in the nuclear area by treatment of heregulin, a glycoprotein that elevates tyrosine phosphorylation of the neu receptor [66]. The rat version of human ErbB-2, p185neu, was first reported to be located in the nucleus, where it is associated with transcriptional activity [67]. A full-length form of nuclear ErbB-2 is involved in COX2 transcriptional regulation via transactivating COX2 gene promoter in breast cancer cells [68]. More recently, it has been shown that nuclear ErbB-2 activates transcription of ribosomal RNA genes through association with RNA polymerase-I and β-actin to ribosomal DNA, leading to increased protein synthesis and cell growth [29]. In addition to nuclear ErbB-2 (p185^ErbB-2^) as an intact molecule, ErbB-2 (p95), which lacks the N-terminal extracellular domain, has also been found in the nucleus [69], where it contributes to acquired therapeutic resistance to ErbB-2 TKIs [70].

#### Nuclear translocation of ErbB-3 and ErbB-4

ErbB-3 exists as a full-length form in the nucleus [24]. Intriguingly, low expression of nuclear ErbB-3 is a predictor of a higher risk of biochemical recurrence in patients with prostate cancer [71,72]. A truncated form of the intracellular domain (ICD) of ErbB-4 undergoing γ-secretase-mediated cleavage has been found in the nucleus of cancer cells [20,73], whereas ErbB-4 has been detected as a full-length receptor in the nuclei of some normal cells [74,75]. The role of nuclear ErbB-4 ICD is still ambiguous but has been shown to be involved in both shorter patient survival [76] and improved patient response to tamoxifen therapy [77] for estrogen receptor-α positive cancers. According to the papers, nuclear cleavable ErbB-4 is associated with shorter survival than cell surface ErbB-4 in the estrogen receptor-positive subset of breast cancer patients, suggesting that the subcellular localization of ErbB-4 is correlated with clinical outcome [76]. On the other hand, researchers have demonstrated that nuclear ErbB-4 ICD acts as a co-activator of estrogen receptor-α and improves patient response to tamoxifen therapy [77]. Additionally, ErbB-4 ICD has been shown to enhance the ubiquitination and degradation of an oncogenic protein, Hdm2, following the increased expression of a tumor suppressor, p53 [78], while blocking Eto2-dependent transcriptional repression involved in cell differentiation [79]. Thus, the controversial role of ErbB-4 ICD as an oncogene or a tumor suppressor requires further systematic investigation.

#### Nuclear translocation of cell surface receptors other than the EGFR family proteins

A substantial body of evidence indicates that several full-length RTKs and cell surface receptors other than EGFR family proteins are translocated to the nucleus, such as fibroblast growth factor receptor (FGFR), vascular endothelial growth factor receptor, insulin-like growth factor-1 receptor, cMet, TrkA, interleukin receptors, interferon-γ receptor, and growth hormone receptors [44,45,80]. Recently, receptor tyrosine kinase-like orphan receptor 1 (Ror1), which belongs to the ROR RTK family, has been shown to be transported to the nucleus through the juxtamembrane domain [81]. In addition, prolactin-mediated nuclear translocation of cell surface prolactin receptor recruits a chromatin-modifying protein to activate Stat5a-driven gene expression [82]. Together, in addition to EGFR family, multiple RTKs have also been detected in the nucleus, raising MRIN as a general phenomenon.

#### Nuclear detection of EGFR family ligands

Ligands of the EGFR family, such as EGF, pro-transforming growth factor-α, and pro-heparin-binding EGF-like growth factor, have been found in the nucleus [83-86]. Schwannoma-derived growth factor, which belongs to the EGF family, can also be detected in the nucleus, where it binds to A + T-rich DNA sequences, leading to a mitogenic response [87]. Furthermore, nuclear translocation of the ICD fragment of neuregulin-1, an ErbB-3/ErbB-4 ligand, results in increased neuronal survival by repressing the cell death response to several regulators [88]. Thus, not only RTKs, ligands can also be translocated into the nucleus, suggesting ligand/receptor association may also occur in the nucleus. In supporting of this notion, the EGF/EGFR complex was indeed detected in the nucleus using the cross-linking experiment between EGF and EGFR [48].

### Molecular and biological functions of nuclear EGFR family proteins

#### Nuclear EGFR family as transcriptional co-activator

**Figure 1:**
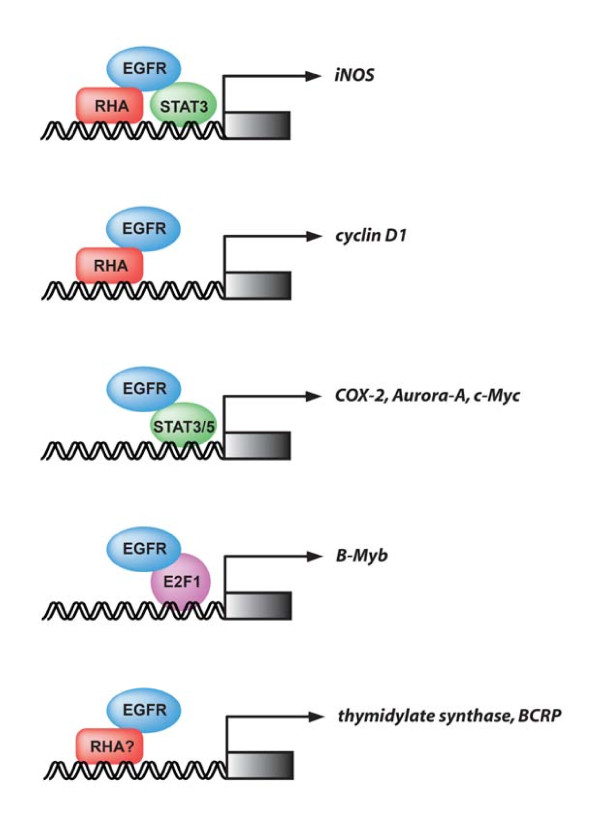
**A summary of nuclear function of EGFR as a transcriptional co-activator.** Nuclear EGFR can function in transcriptional regulation to enhance expression levels of target genes, including iNOS (**A**), cyclin D1 (**B**), COX-2 (**C**), Aurora-A (**C**), c-Myc (**C**), B-Myb (**D**), thymidylate synthase (**E**), and BCRP (**E**), through activation of transcriptional factors, such as STAT and E2F1. EGFR also associates with RHA in the nucleus, where the EGFR/RHA complex binds to the target gene promoter, including iNOS (**A**) and cyclinD1 (**B**), through the recruitment of RHA to the ATRS of the target gene promoter to mediate its transcriptional activation. In addition to RHA, EGFR is also recruited to the iNOS gene promoter through STAT3 to the STAT3-binding site (**A**). Whether RHA is involved in the nuclear EGFR-mediated activation of thymidylate synthase and BCRP (**E**) has not yet been explored.

Members of the nuclear EGFR family that contain an intrinsic transactivation activity at the C-terminal acidic region, including EGFR, ErbB-2, and ErbB-4 [20,48,67,68], can function in transcriptional regulation to enhance target gene expression through activation of transcriptional factors. Several gene promoters have been identified as the targets of the nuclear EGFR family receptors (Figure 1), such as cyclin D1 [48], B-Myb [89], iNOS [90], Aurora-A [91], COX-2 [30], c-Myc [14], thymidylate synthase [63], and BCRP [17], which are involved in tumorigenesis, chromosome instability, and chemo-resistance. Upon EGF stimulation, activated nuclear EGFR acts as a transcriptional co-activator, binding to an AT-rich response sequence (ATRS) of the cyclin D1 promoter and stimulating cyclin D1 expression [48]. A recent paper identified potential nuclear EGFR interacting proteins using an unbiased mass spectrometry approach: it showed that RNA helicase A (RHA) associates with nuclear EGFR and the EGFR-RHA complex activates cyclin D1 transcription through binding of RHA to the ATRS [92]. MUC1 has also been found to interact with nuclear EGFR and promote EGFR-mediated cyclin D1 gene expression [11]. Moreover, activated nuclear EGFR has been shown to bind to the ATRS motif and promote B-Myb, iNOS, COX-2, and Aurora-A genes through interaction with transcription factors, such as E2F1, STAT3, and STAT5A, respectively [30,89-91]. Recently, researchers demonstrated that EGFR is transported to the nucleus through serine phosphorylation by Akt, and the nuclear EGFR then targets multiple ATRSs on the BCRP promoter in gefitinib-resistant cells, which is involved in chemo-resistance [17,93]. In addition, nuclear EGFR and HER2 activate thymidylate synthase gene transcription via binding to its promoter, and this interaction between EGFR/HER2 with thymidylate synthase promoter is blocked by a dual EGFR/HER2-TKI, lapatinib [63]. A novel nuclear complex including EGFR together with c-Src kinase and STAT3 can associate with the c-Myc promoter in pancreatic cancer, suggesting that this heteromeric complex may regulate the c-Myc gene [14]. Nuclear EGFRvIII has also been found to cooperate with STAT3 to activate COX-2 gene expression in glioblastoma cells, resulting in glioma tumorigenesis [26,30]. In line with the studies of nuclear EGFR, nuclear ErbB-2 can transactivate COX2 gene expression through binding to a specific DNA element, the HER2-associated sequence, within the promoter, whereas the transcriptional factors involved remain to be identified [68]. In addition, the ICD of ErbB4 translocates to the nucleus upon ligand stimulation and associates with STAT5A to transactivate the β-casein gene promoter [94]. Nuclear ErbB-4 ICD has been shown to interact with Eto-2, a nuclear corepressor in breast cancer, and block Eto-2-dependent transcriptional repression [79]. A recent report characterizing EGFR as a DNA-binding protein using unbiased approaches [95] further supports the notion that the nuclear EGFR family plays a role in transcriptional regulation.

#### Nuclear EGFR family as protein kinase involving protein-protein interaction

Nuclear EGFR family receptors, except ErbB-3, maintain tyrosine kinase activity. Nuclear EGFR associates with and phosphorylates the chromatin-bound form of proliferative cell nuclear antigen (PCNA), which stabilizes PCNA protein, leading to DNA replication and DNA damage repair [19]. A series of studies showed that DNA damage pathways, such as those activated by ultraviolet irradiation or cisplatin treatment, can induce the interaction between nuclear EGFR and DNA-dependent protein kinase (DNA-PK) [15,50,60], which is a central enzyme of the nonhomologous end-joining repair of DNA double-strand breaks, contributing to DNA repair and chemo- and radio-resistance. It is not yet clear but worthwhile to determine whether EGFR phosphorylates DNA-PK to regulate its activity. Moreover, ErbB-2 is able to co-localize with the cyclin-dependent kinase p34^Cdc2^ in both the cytoplasm and the nucleus, and subsequently phosphorylate it, leading to resistance to taxol treatment in breast cancer [96], suggesting that ErbB-2 functions as a kinase in the nucleus. Furthermore, ErbB-4 ICD fragment has been shown to interact with and phosphorylate the nuclear protein Hdm2, and consequently enhance Hdm2 ubiquitination, increase p53 transcriptional activity using a p21 luciferase reporter, and increase p53 and p21 expression [78].

### EGFR subcellular trafficking from the cell surface to different compartments

**Figure 2:**
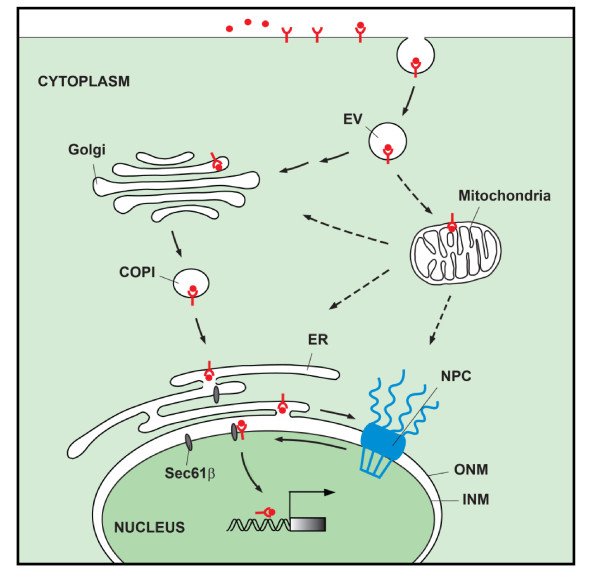
**A diagram of the EGFR family receptors trafficking to different compartments.** The endocytic vesicles carrying EGFR can be transported from the cell surface to several intracellular organelles, including the Golgi apparatus, the ER, the mitochondria, and the nucleus. It has been documented recently that COPI vesicle-mediated retrograde transport from the Golgi to the ER is involved in the EGFR nuclear trafficking. Integral EGFR inserted into the ER membrane is targeted to the INM of the nuclear envelope (NE) through the ONM and NPC via a model of integral trafficking from the ER to the NE transport (INTERNET). The INM-embedded EGFR can be released from the lipid bilayer to the nucleoplasm within the nucleus by the association with the translocon Sec61β located in the INM. In addition to the nuclear import of cell surface EGFR, the internalized EGFR can also be trafficked to the mitochondria; however, the molecular mechanism underlying the cell surface-to-mitochondria trafficking of EGFR remains unclear. Whether the localization of EGFR in the mitochondria is involved in the EGFR trafficking to the Golgi, the ER, and the nucleus has not yet been explored. The scale of the diagram does not reflect the relative sizes of different molecules or subcellular structures. EV, endocytic vesicle; COPI: coat protein complex I; NPC, nuclear pore complex; ER, endoplasmic reticulum; ONM, outer nuclear membrane; INM, inner nuclear membrane.

The signaling duration and intensity of transmembrane RTKs stimulated by extracellular ligands is regulated by receptor endocytosis, which is characterized as a membrane and vesicular trafficking process. After ligand-induced endocytosis, cargo proteins carried in budding vesicles can be delivered from donor membranes to acceptor subcellular organelles through fusion pathways, by which RTKs and their cognate ligands are internalized into cytoplasmic vesicles and sequentially removed from the cell surface [97,98]. Accumulating evidence suggests that the internalized EGFR embedded within the early endosomes has several potential destinations via endosomal sorting. First, EGFR can be recycled back to the cell surface through either the recycling endosomes or a direct recycling pathway. Second, EGFR can be sorted into the late endosomes and subsequently degraded by lysosomes. In addition to the above well-characterized trafficking routes, a novel mode of the EGFR signaling pathway, in which EGFR after endocytosis can be transported from the cell surface to different compartments within cells, including the Golgi apparatus, the ER, the mitochondria, as well as the nucleus, has also been reported [25,27,44] (Figure 2). We will highlight the subcellular trafficking mechanism of EGFR in the following paragraphs.

#### Vesicle trafficking of EGFR to the Golgi apparatus and to the ER

Cargo proteins carried in budding vesicles after endocytosis can be sorted to the biosynthetic/secretory compartments, such as the Golgi apparatus and the ER, known as retrograde transport, which is involved in multiple cellular functions. The retrograde route from early endosomes to the Golgi apparatus occurs in several mammalian cargo proteins [99] while that to the ER is commonly used by exogenous viruses and toxins [100]. Recently, researchers identified the first example of retrograde trafficking, in which EGFR is transported from the Golgi to the ER to regulate the nuclear transport of cell surface EGFR RTK [37]. Upon EGF treatment, full-length EGFR anchors to the membranes of the Golgi and the ER, with the C-terminus exposed to the cytoplasm and the N-terminus masked inside the Golgi and ER lumen [37]. Another group of researchers reported that EGFR is trafficked from the cell surface to the ER in response to EGF [32]. It has also been well-documented that coat proteins, including coat protein complex I (COPI) involved in the Golgi-to-ER retrograde transport and COPII in the ER-to-Golgi anterograde transport, play a central role in vesicular transport to target its intracellular destination [101-103]. Wang et al. [37] reported that γ-COP, one of the subunits of the COPI coatomer, associates with EGFR and mediates EGF-dependent EGFR nuclear transport. Together, these findings suggest that endosomal membrane-embedded cell surface EGFR in a membrane-associated environment travels from the cell surface to the nucleus within the Golgi and ER membranes via COPI-mediated vesicle trafficking. Most recently, an interesting report showed that COPI acts not only in vesicular retrograde transport but also, unexpectedly, in tubular formation, which is involved in anterograde intra-Golgi transport [104]. It would be interesting to determine the physiological roles of COPI tubules to further expand our knowledge on how the COPI complex regulates subcellular cargo sorting.

#### EGFR trafficking to the mitochondria

Upon EGF treatment, full-length EGFR anchors to the mitochondria, where it associates with cytochrome c oxidase subunit II, a key component of the oxidative phosphorylation cascade in regulating apoptosis through cytochrome c release from the mitochondria [105,106]. In addition, clathrin-mediated endocytosis, c-Src kinase activity, and a putative mitochondrial localization signal within the juxtamembrane domain of EGFR are involved in EGFR translocation to the mitochondria [106]. The alternate subcellular localization of EGFR to the mitochondria may contribute to cellular survival in modulating cytochrome c oxidase subunit II-dependent mitochondrial functions. A recent paper showed that both EGFR and EGFRvIII can be translocated to the mitochondria after treatment of apoptosis inducers and an EGFR-tyrosine kinase inhibitor, and the mitochondrial targeting of these receptors is responsible for drug resistance [107]. Furthermore, cetuximab has also been shown to induce mitochondrial accumulation of EGFRvIII [108], suggesting that mitochondrial EGFR/EGFRvIII plays a role in therapeutic response to EGFR-targeting drugs.

However, how EGFR is transported to the mitochondria remains unclear. Further study is required to determine whether EGFR is integrated into the mitochondrial membrane through endosomal membrane fusion with the mitochondria or via other potential pathways.

#### EGFR trafficking to the nucleus

EGFR family receptors have been discovered to be transported to the nucleus, where they exist as full-length or truncated forms and exert a number of functions, as described in the previous sections (Table 1). However, the trafficking mechanism for the nuclear transport of endosome-embedded EGFR family has been overlooked for decades. In addition to the recent paper reporting that COPI-mediated retrograde trafficking regulates nuclear translocation of EGFR [37], there have reports that identified the putative nuclear localization signals (NLSs) within all of the EGFR family members [24,30,68,90,94]. Researchers have further characterized a tripartite NLS of EGFR, which is different from the traditional mono- and bipartite NLSs, contains three clusters of basic amino acids, and is conversed within the juxtamembrane regions among the EGFR family [109]. It has been demonstrated that NLS and importin-β are involved in the nuclear translocation of EGFR and ErbB-2 [110,111], in which importin-β forms a complex with NLS-harboring molecules and is responsible for nuclear translocation through binding to the nucleoporins of nuclear pore complexes. Furthermore, receptor endocytosis and endosomal sorting through association with early endosomal markers in the nucleus are also required for nuclear translocation of EGFR and ErbB-2 [110,111]. Interestingly, in addition to its localization in the nucleoplasm within the nucleus, ErbB-2 has been observed specifically in the nucleolus, where it associates with RNA polymerase-I [29]. Further investigations of the potential trafficking mechanism of ErbB-2 to the nucleolus and of the identity of EGFR family receptors other than ErbB-2 are warranted. Moreover, the exportin CRM1 has been shown to be involved in the nuclear export of cell surface RTKs, including EGFR, ErbB-2, and ErbB-3, although their nuclear export signals have not yet been identified [24,110,111].

**Table 1:** Existence of EGFR family receptors in the nucleus

**EGFR family receptor**	**Type**	**Size (kDa)**	**Cell/Tissue detection**	**Reference**
EGFR	Full-length	170	hepatocyte, breast, pancreatic, head and neck, glioblastoma, lung, and skin cancers, etc.	[12,14,22,28,30,38,39,49]
EGFRvIII	In-frame deletion	145	normal glial cells, glioblastoma, prostate cancer	[26,30,65]
EGFR variant mLEEK	In-frame splice	45	glioblastoma	[16]
ErbB-2: p185^ErbB-2^	Full-length	185	breast cancer	[29,68,110]
ErbB-2: p95^ErbB-2^	N-terminal truncation	95	breast cancer	[69,70]
ErbB-3	Full-length	185	nonmalignant epithelial cells, breast cancer, prostate cancer	[24,65,71,72]
ErbB-4	Full-length	180	normal cells: brain cells, endothelial cells	[74,75]
ErbB-4 ICD	N-terminal truncation	80	breast cancer, prostate cancer, embryonic brain	[20,65,73,76]

#### Trafficking of EGFR from the inner nuclear membrane to the nucleoplasm

EGFR appears to be present in the inner nuclear membrane (INM) or nuclear matrix [112,113]; however, the exact trafficking mechanism in INM translocation is unclear. Recently, it has been shown that, upon EGF stimulation, cell surface EGFR is targeted to the INM through a mechanism termed INTERNET, which stands for the integral trafficking from the ER to the nuclear envelope transport, pathway [38,44]. Furthermore, the INM-anchored EGFR has been proposed to be extracted from the INM to the nucleoplasm by a translocon Sec61β-dependent process, in which Sec61β, traditionally associated with the ER, displays a previously unrecognized location and role in regulating EGFR nuclear transport via the association with EGFR in the INM [38]. The newly identified Sec61β function provides a plausible explanation for how the membrane-bound cell surface EGFR remains in a membrane-associated environment while it is translocated from the lipid bilayer of the INM to the nucleus. But beyond our preliminary understanding of the Sec61β-associated pathway in the nucleus, the trafficking mechanism remains largely unexplored. It is worthwhile to mention that whether the intra-nuclear EGFR represents as a soluble receptor free of membrane is still unclear; therefore, one possible mechanism raised is that an endocytosis-like mechanism in the nuclear envelope transports EGFR from the INM to the nucleoplasm, where EGFR remains nuclear membrane-embedded. A more systematic study is required to further address this hypothesis. Interestingly, Sec61β, which is traditionally thought to be localized in the ER, has been proposed to extract EGFR from lipid layers of the ER membrane for delivery to the cytoplasm via ER-associated degradation pathway. At that point, cytoplasmic EGFR can be transported to the nucleus through the association of importin-β [32]. However, this Sec61β-mediated ER-associated degradation model in regulating EGFR nuclear trafficking needs to be further verified since researchers could not detect EGFR in the cytoplasm in EGF-treated cells [32].

## Conclusions

Multiple integral membrane proteins, including all members of EGFR family, have been reported to function in the nucleus. Recently, researchers discovered a logical route for the nuclear translocation of EGFR in response to EGF, in which cell surface EGFR travels to the nucleus, all the way in a membrane-bound environment, through the Golgi-to-ER retrograde pathway and INTERNET model to the INM in the nucleus [37,38]. The major questions yet to be investigated include at least the following: First, how is EGFR embedded in the endosomal membrane shuttled to the Golgi apparatus? One proposal is that the small GTPase protein Rab7b is essential for retrograde trafficking from the endosomes to the Golgi [114]. Whether specific Rab proteins are involved in EGFR trafficking to the nucleus needs to be determined. Second, does membrane-bound trafficking serve as a general mechanism for nuclear transport of other RTKs and cell surface receptors? Since FGFR-1 has an atypical transmembrane domain, which functions not only as a transmembrane RTK but also as a soluble cytoplasmic protein [115-117], unlike the EGFR family proteins, it may be useful to compare the trafficking mechanism of FGFR-1 nuclear translocation with that of EGFR. Last, does subcellular trafficking of EGFR to different compartments contribute to their different roles? Investigating systematically how cell surface RTKs are transported to various destinations will advance our knowledge of their unique functions of RTKs in different cellular compartments. Since many of these RTKs are therapeutic targets, the areas of research may have important clinical implication.

## Abbreviations

RTKs = receptor tyrosine kinases; EGFR = epidermal growth factor receptor; STAT = signal transducer and activator of transcription; TKIs = tyrosine-kinase inhibitors; MRIN = membrane receptors in the nucleus; ER = endoplasmic reticulum; BCRP = breast cancer-resistant protein; COX-2 = cyclooxygenase-2; FGFR = fibroblast growth factor receptor; ATRS = AT-rich response sequence; RHA = RNA helicase A; PCNA = proliferative cell nuclear antigen; DNA-PK = DNA-dependent protein kinase; COPI = coat protein complex I; NLSs = nuclear localization signals; INTERNET = integral trafficking from the ER to the nuclear envelope transport; ERAD = ER-associated degradation.

## Competing interests

The authors declare no conflict of interests.

## Authors’ contributions

Y-NW and M-CH outlined the manuscript. Y-NW wrote the draft version and M-CH finalized the manuscript. Both authors read and approved the final manuscript.
